# Impact of long‐term management with sleep medications on blood pressure: An Australian national study

**DOI:** 10.1002/brb3.2943

**Published:** 2023-04-03

**Authors:** Mumtaz Begum, David Gonzalez‐Chica, Carla Bernardo, Nigel Stocks

**Affiliations:** ^1^ Discipline of General Practice, Adelaide Medical School Faculty of Health and Medical Science, The University of Adelaide South Australia Australia; ^2^ Adelaide Rural Clinical School Adelaide Medical School Faculty of Health and Medical Science The University of Adelaide South Australia Australia

**Keywords:** average treatment effect, benzodiazepines, blood pressure, electronic health records, primary care, sleep medication

## Abstract

**Background:**

There is mixed evidence about the impact of long‐term management with hypnotic medications on blood pressure (BP).

**Aim:**

To estimate the effect of short‐ and long‐term management with benzodiazepine and z‐drugs (BZD) on BP.

**Method:**

Open cohort study using deidentified electronic health records of 523,486 adult regular patients (42.3% males; mean age 59.0 ± 17.0 years) annually attending 402 Australian general practices between 2016 to 2018 (MedicineInsight database). Average treatment effects (ATE) of recorded incident BZD prescriptions in 2017 on systolic (SBP) and diastolic (DBP) BP after starting these prescriptions were computed using augmented inverse probability weighting (AIPW).

**Results:**

In 2017, 16,623 new cases of short‐term management with BZD and 2532 cases of long‐term management with BZD were identified (incidence 3.2% and 0.5%, respectively). The mean BP among those not treated with BZD (reference group) was 130.9/77.3 mmHg. Patients prescribed short‐term BZD showed a slightly higher SBP (ATE 0.4; 95% CI 0.1, 0.7) and DBP (ATE 0.5; 95% CI 0.3, 0.7), while those on long‐term BZD prescriptions showed lower SBP (ATE ‐1.1; 95% CI −2.0, −0.2), but no effect on DBP (ATE −0.1; 95% CI −0.8, 0.5). However, long‐term BZD prescriptions showed a stronger BP‐lowering effect among patients aged 65+ years (SBP ATE −2.5 [95% CI −3.8, −1.3]; DBP ATE −1.0 [95% CI −1.7, −0.2]), but almost no effect was observed among younger patients.

**Conclusion:**

Long‐term management with BZD had a BP‐lowering effect among older patients. These findings add new evidence to current recommendations on limiting long‐term BZD management in the elderly.

## Graphical abstract

- There was a strong BP‐lowering effect associated with long‐term management with BZD among older patients (65+ years), with 2.5 mmHg reduction in the mean SBP and 1.0 mmHg reduction in the mean DBP, compared with those not managed with BZD.
- The average effect of short‐term BZD management on BP in this age group was tenuous.
- Our findings add evidence to current recommendations about the cautious use of long‐term BZD among older patients, as their BP‐lowering effect can further increase the risk of dizziness and falls in this vulnerable population.

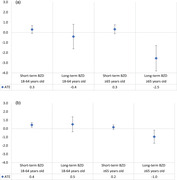

## 1 INTRODUCTION

Long‐term benzodiazepine and z‐drugs (BZD) use have a substantial individual and economic burden as they can increase the risk of dependence, cognitive dysfunction, impaired quality of life, hospitalizations, and deaths (Mathieu et al., 2021; Parsaik et al., 2016; Soyka, 2017). Indeed, a meta‐analysis of 10 studies showed a 60% increased risk of mortality among benzodiazepine users compared with nonusers, with a similar effect size observed among z‐drug users (Parsaik et al., 2016). In Australia, benzodiazepines remain the second most common group of drugs (behind opioids) involved in drug‐related deaths, being responsible for 41.6% of all unintentional drug‐induced fatalities in 2018 (Penington Institute, 2020).

Despite current recommendations of short‐term use only (The Royal Australian College of General Practitioners, 2015), BZD are commonly used long‐term as hypnotics and/or anxiolytics, especially among older people (Holliday et al., 2017; Woods et al., 2022; Zheng et al., 2020). Higher BZD use in the elderly is particularly concerning, as it has been related to falls, fractures, loss of independence, and hospitalizations (Madhusoodanan & Bogunovic, 2004; Poly et al., 2020; Treves et al., 2018). The increased risk of falls among BZD users is attributed to the BZD sedative effect that compromises gait and balance, as well as cognitive and psychomotor functioning impairment related to the use of these drugs (de Groot et al., 2013; Ng et al., 2018). Moreover, apart from the hypnotic‐sedative and muscle‐relaxant effects, BZDs have vasodilator, vasorelaxant and myorelaxant properties that may reduce blood pressure (BP) levels (Colussi et al., 2017; Kagota et al., 2021), making older people more vulnerable to falls and accidents. In fact, hypotension is a well‐known risk factor for falls and accidents in old age (Klein et al., 2013; Sagawa et al., 2018). Some studies have demonstrated the hypotensive effect of short‐term use of single benzodiazepines (e.g., temazepam, diazepam) (Bosone et al., 2018; Kitajima et al., 2004). However, the evidence is inconsistent, as other studies found the opposite effect (Fogari et al., 2019). Most of these studies investigated small samples, analyzed the effect of one benzodiazepine only, and/or focused on short‐term use only. Hence, there is a paucity of evidence about the impact of long‐term BZD use on BP.

Only three international studies explored the association between long‐term BZD use and BP, reporting inconsistent findings (Hein et al., 2019; Mendelson et al., 2017; Rivasi et al., 2020). One of the studies used retrospective data from a large regional hospital database in Israel (*n* = 670 BZD treated, period 2009–2015) and found lower systolic and diastolic BP among long‐term BZD users than nonusers (up to 2.1 mmHg and 3.2 mmHg lower BP, respectively) (Mendelson et al., 2017). An Irish study found that older patients regularly treated with benzodiazepines (*n* = 33) had a systolic BP 10 mmHg lower than nonusers (*n* = 505) (Rivasi et al., 2020). On the contrary, a Belgium retrospective study using data of 1272 adults with insomnia (single hospital data, period 2002–2014) reported that long‐term use of short/intermediate‐acting benzodiazepines was associated with a two‐fold increased risk of hypertension (Hein et al., 2019). However, long‐term use of long‐acting BZD had an opposite effect on that outcome. The discrepancies between these studies could result from sample characteristics, differences in the adjustment for potential confounders, and the differentiation between long‐term exposure to short‐acting (elimination half‐life ≤24 h) or long‐acting BZD (half‐life > 24 h).

Therefore, we used a large Australian national primary care database (MedicineInsight) with a wide range of information on potential confounders to investigate the longitudinal effect of short‐ and long‐term incident management with BZD on average BP levels after starting these medications, as well as the heterogeneity of these relationships according to the patients’ age and elimination half‐life of these drugs. To achieve this objective, we estimated the average treatment effect (ATE) of incident BZD on BP levels using augmented inverse probability of weighting (AIPW). AIPW deals with measured confounding (in a counterfactual approach) by creating a pseudo population where, every individual is considered as both, exposed (BZD user) and unexposed (BZD nonuser) (Funk et al., 2011; Hernán & Robins, 2016). The advantage of that technique over traditional regression models is that, in the absence of unmeasured confounding, it provides results that allow similar interpretation as findings from a randomized trial (Funk et al., 2011).

## 2 METHOD

### 2.1 Study design and population

MedicineInsight is a national primary care database that, in 2018, comprised over 2700 general practitioners (GP) from 662 general practices (8.2% of all practices in Australia), with available data since 2011 (Busingye et al., 2019). Deidentified electronic health records (EHR) are extracted monthly from participating practices, including information on sociodemographic characteristics, clinical measurements, diagnoses, pathology results and prescribed medications.

To improve data consistency, only practices without a gap of more than 6 weeks in the previous two years in data provision and with a consistent number of consultations over time (i.e., ratio lower than five between the maximum and minimum number of annual consultations in each practice) were included. Only one recorded visit per day per patient was counted, and administrative contacts (e.g., phone calls, reminders) were excluded. We used deidentified EHR of patients aged 18+ years attending these general practices between 2016 and 2018. Sample was restricted to regular patients (who had at least one consultation per year between 2016 and 2018) to ensure that they would have available data on the exposure in 2016 and 2017, and the outcome in 2018.

### 2.2 Exposure (incident short‐ and long‐term BZD management)

**FIGURE 1:**
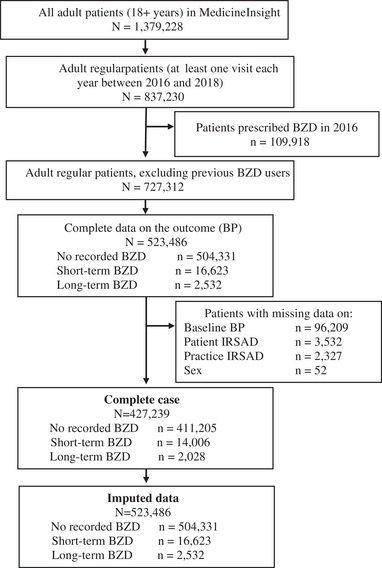
Flowchart of the study sample. Note. BZD: benzodiazepines and z‐drugs; BP: blood pressure; IRSAD: The Index of Relative Socio‐economic Advantage and Disadvantage.

The exposure of interest was incident short‐ and long‐term BZD management in 2017. Data on BZD prescriptions was extracted from the field script item (Busingye et al., 2019) using generic and brand names of all benzodiazepines (temazepam, diazepam, nitrazepam, oxazepam, lorazepam, clonazepam, alprazolam, flunitrazepam, midazolam, clobazam, bromazepam) and z‐drugs (zopiclone and zolpidem) approved for use in Australia (Begum et al., 2021; The Royal Australian College of General Practitioners, 2015). Long‐term incident BZD management was defined as the provision of at least three BZD scripts within 180 days, starting in 2017, with the second script provided by the GP after 28 days from the initial script (The Royal Australian College of General Practitioners, 2015; Woods et al., 2022). An episode of long‐term BZD prescribing ended when the patient had not received any new BZD prescription for benzodiazepine or z‐drug for 180 or more days. All other situations where a patient started BZD in 2017 were considered short‐term incident management. Only the first recorded episode of BZD management (i.e., either short‐ or long‐term) in 2017 was considered for analysis, and all patients who had a prescription of BZD in 2016 were excluded of our sample. The exposure was then classified as (i) no BZD exposure in 2017; (ii) short‐term incident management with BZD in 2017; (iii) long‐term incident management with BZD in 2017. Therefore, out of the 1,379,228 adult patients in the MedicineInsight database, the final sample consisted of 523,486 regular patients who had no recordings of BZD prescriptions in 2016 (Figure 1).

For sensitivity analyses, long‐term incident BZD management was further classified based on the half‐life of the prescribed drugs (long‐acting BZD [half‐life > 24 h]: diazepam, nitrazepam, clonazepam, clobazam and flunitrazepam; short‐intermediate acting BZD [half‐life ≤24 h]: temazepam, oxazepam, lorazepam, bromazepam, alprazolam, midazolam, zolpidem, and zopiclone). Depending on the type of BZD received, long‐term incident BZD management was subclassified as (1) only short‐intermediate acting BZD, (2) only long‐acting BZD, and (3) a mix of both short‐intermediate and long‐acting BZD.

### 2.3 Outcome (systolic and diastolic blood pressure)

We used data from 2016 to extract baseline information on BP levels for adjustment. As all patients had multiple BP measurements, the baseline was defined as the median of all BP measurements recorded between January and December 2016.

Data from 2017–2018 was used to investigate BP levels as the primary outcome of the study. For those not managed with BZD in 2017 (i.e., the reference group), the outcome was defined as the median of all BP measurements recorded between January 2017 and December 2017. For those exposed to short‐term BZD, the outcome was defined as the first BP measurement taken at least 28 days after the start of the short‐term BZD episode in 2017. For those exposed to long‐term BZD, the outcome was defined as the median BP between the first BZD script in 2017 and the end of the long‐term BZD episode (2017 or 2018).

### 2.4 Confounding

Information on potential confounders was identified a priori based on evidence from the literature, as they have been associated with higher BZD prescription rates and alterations in BP levels (Begum et al., 2021; Hein et al., 2019; Mendelson et al., 2017; Pan et al., 2015; Rivasi et al., 2020). The relationship between these variables was presented graphically using a directed acyclic graph (Supplementary Figure S1). All data about potential confounders was sourced from the MedicineInsight database (Busingye et al., 2019). General practice characteristics included the rurality (major cities, inner regional, outer/remote/very remote area) and the Index of Relative Socio‐economic Advantage and Disadvantage (IRSAD in quintiles, grouped as advantaged/highest two quintiles, middle, and disadvantaged/lowest two quintiles) of the practice. IRSAD is a macrolevel indicator of socioeconomic position based on postcodes and developed by the Australian Bureau of Statistics to consider a range of socioeconomic variables (Australian Bureau Of Statistics, 2018). Patients characteristics included age (18–34 years, 35–49 years, 50–64 years, 65–74 years, ≥75 years), sex (male, female), Aboriginal and Torres Strait Islander peoples (yes, no, not stated/not recorded), patient's IRSAD (grouped as for practice IRSAD), most recent smoking status (nonsmokers, ex‐smokers, smokers), baseline BP (2016), and clinical history data recorded in 2016 or 2017 on the use of antihypertensive medication (AHT yes/no, based on prescribed medications), mental stress (yes/no, including conditions such as stress, depression, anxiety or post‐traumatic stress disorder), diabetes diagnosis (yes/no), and sleep issues/insomnia (yes/no) (Ali et al., 2020; Thomas & Calhoun, 2017) recorded as a diagnosis or reason for encounter. Methods used to extract clinical history data has been reported elsewhere (Roseleur et al., 2021; Woods et al., 2022). Due to the large amount of missing data for the body mass index (BMI, 59%), that variable was only included in sensitivity analysis.

### 2.5 Statistical analyses

In this study, the “treatment” group was defined as those newly managed with BZD (short‐term or long‐term in 2017). In primary analyses, we estimated the ATE of short‐term or long‐term incident BZD management on BP, compared with BZD nonuser, using AIPW (Funk et al., 2011; Hernán & Robins, 2016). AIPW is a doubly robust method that yields unbiased estimates if at least one of the models is correctly specified (Funk et al., 2011). The two models included in this study were (i) the treatment model, used to compute the probability of short‐ or long‐term incident BZD management given the observed confounders (multinomial logit regression [Stata, manual]), and (ii) the outcome model (linear regression). The reciprocal of the probability obtained in the treatment model was used as the weight in the outcome model. Except for BMI, all confounding variables specified above and in the directed acyclic graph were included in both models. In addition, the number of consultations in 2017 was included in the treatment model.

Considering that BZD use increases with age (Woods et al., 2022) and older groups are more susceptible to the side effects of these drugs (Madhusoodanan & Bogunovic, 2004; Poly et al., 2020; Treves et al., 2018), we also computed the ATE of BZD on BP stratified by age (18–64 years or ≥65 years) using the aforementioned methodological approach.

All analyses were repeated considering traditional linear regression models to provide comparable estimates with previous studies in the investigation of the effects of short‐ and long‐term BZD on BP. Finally, we run sensitivity analyses considering the ATE of long‐term incident BZD management on BP according to the type of BZD received (only short‐intermediate acting BZD, only long‐acting BZD, or a mix of both short‐intermediate and long‐acting BZD compared to BZD nonuser). In addition, we also conducted sensitivity analyses excluding patients with diagnosed obstructive sleep apnea.

### 2.6 Missing data

Missing data was less than 1% for all the covariates included in this study, except baseline BP (18.4%) (Figure 1). We used multiple imputation by chained equation to impute the missing data on confounders. Multiple imputation was conducted to account for the potential bias if the association varies between patients with and without complete data (Sterne et al., 2009). Twenty data sets were generated, and all confounders mentioned in Table 1 were included in the imputation model. In addition, number of consultations in 2017 and the practice identification were also included as auxiliary variables to inform the imputation model. We computed the mean ATE, within imputation variance and between imputation variance; and combined the estimates from the 20 imputed data sets using Rubin's rule (Rubin, 2004).

**TABLE 1:** Sample distribution of regular patients who visited Australian general practice between 2016 and 2018 according to sociodemographic and clinical characteristics by management with BZD (imputed data, total *N* = 523,486)

	No BZD	Short‐term BZD	Long‐term BZD
	*n* = 504,331	*n* = 16,623	*n* = 2532
	% (95% CI)	% (95% CI)	% (95% CI)
**Practice characteristics**			
Practice IRSAD			
Advantaged, highest two quintiles	40.1 (34.4, 45.9)	44.3 (38.2, 50.4)	38 (31.7, 44.3)
Middle	24.1 (18.9, 29.4)	23.1 (17.9, 28.4)	23.9 (18.3, 29.5)
Disadvantaged, lowest two quintiles	35.6 (30, 41.2)	32.4 (26.8, 38.1)	37.9 (31.5, 44.3)
GP remoteness			
Major cities	57.8 (51.9, 63.6)	60.5 (54.5, 66.4)	55.9 (49.4, 62.4)
Inner regional	28.3 (22.8, 33.7)	27 (21.5, 32.5)	28.8 (22.9, 34.7)
Outer/remote/very remote	13.8 (9.9, 17.7)	12.3 (8.5, 16.1)	15.1 (10.4, 19.9)
**Patient's characteristics**			
**Gender**			
Male	42.6 (42, 43.2)	35 (33.9, 36)	40.7 (38.6, 42.8)
Female	57.3 (56.7, 57.9)	64.9 (63.9, 66)	59.2 (57.1, 61.3)
Age			
18–34 years	12.6 (12, 13.1)	9.2 (8.4, 9.9)	7.9 (6.8, 9)
35–49 years	17.6 (16.9, 18.3)	16.9 (16, 17.8)	18.4 (16.8, 20)
50–64 years	28.7 (28.3, 29.1)	28.2 (27.3, 29)	23.6 (21.9, 25.3)
65–74 years	22.1 (21.6, 22.7)	23.9 (23, 24.8)	20.8 (19.1, 22.4)
75+ years	18.8 (18, 19.6)	21.6 (20.6, 22.7)	29 (26.8, 31.3)
**Patient IRSAD**			
Advantaged, highest two quintiles	39.4 (34.8, 44)	42.9 (38.1, 47.8)	35.9 (30.9, 41)
Middle	23.7 (20, 27.4)	23.1 (19.2, 27)	24.2 (19.9, 28.5)
Disadvantaged, lowest two quintiles	36.8 (32.2, 41.4)	33.8 (29.1, 38.5)	39.7 (34.4, 45)
**Insomnia/sleep issues**			
No	97.8 (97.7, 98)	78.7 (77.3, 80.1)	64.8 (62.3, 67.3)
Yes	2.1 (1.9, 2.2)	21.2 (19.8, 22.6)	35.1 (32.6, 37.6)
**AHT medications**			
No	53 (52, 54)	45.6 (44.3, 46.8)	42.3 (40.1, 44.4)
Yes	46.9 (45.9, 47.9)	54.3 (53.1, 55.6)	57.6 (55.5, 59.8)
**Diabetes**			
No	90.8 (90.3, 91.2)	89.7 (89, 90.5)	88.3 (86.9, 89.6)
Yes	9.1 (8.7, 9.6)	10.2 (9.4, 10.9)	11.6 (10.3, 13)
**Mental stress**			
No	90.5 (90, 90.9)	66.8 (65.2, 68.3)	55.1 (52.7, 57.5)
Yes	9.4 (9, 9.9)	33.1 (31.6, 34.7)	44.8 (42.4, 47.2)
**Smoking**			
Nonsmokers	56.6 (55.7, 57.5)	52 (50.8, 53.2)	42.4 (40.3, 44.5)
Smoker	9.7 (9.2, 10.2)	11.7 (10.9, 12.5)	19.2 (17.5, 21)
Ex‐smokers	29.1 (28.4, 29.7)	32.2 (31.1, 33.2)	34 (32.1, 35.8)
Not stated/not recorded	4.5 (3.9, 5)	3.9 (3.3, 4.6)	4.2 (3.2, 5.3)
**Aboriginal and Torress Strait Islanders**			
Neither Aboriginal nor Torres Strait Islander	80.9 (78.1, 83.7)	81.7 (78.6, 84.9)	81.1 (77.7, 84.5)
Aboriginal and/or Torres Strait Islander	1.7 (1.4, 1.9)	2.1 (1.6, 2.5)	3 (2.1, 3.8)
Not stated	17.3 (14.5, 20.2)	16 (12.8, 19.3)	15.8 (12.4, 19.2)
**Basleine DBP**	77.5 (77.4, 77.5)	77.7 (77.6, 77.9)	77.4 (77, 77.9)
**Basleine SBP**	131.9 (131.8, 131.9)	131.1 (130.8, 131.3)	130.9 (130.3, 131.6)
Temazepam		39.7 (38.3, 41.1)	32.9 (30.7, 35.2)
Diazepam		38.8 (37.5, 40)	31.1 (29, 33.3)
Oxazepam		7.7 (6.9, 8.5)	13.4 (11.7, 15.2)
Nitrazepam		1.1 (0.9, 1.3)	2.7 (1.9, 3.5)
Alprazolam		1.7 (1.4, 1.9)	2.8 (2.1, 3.5)
Lorazepam		2.5 (2.1, 2.8)	3.3 (2.6, 4.1)
Clonazepam		1.3 (1.1, 1.5)	1.2 (0.8, 1.7)
Flunitrazepam		0 (0, 0.1)	0.1 (0, 0.2)
Clobazam		0.1 (0, 0.1)	0.1 (0, 0.3)
Bromazepam		0.2 (0.1, 0.3)	0.2 (0, 0.4)
Zolpidem		3 (2.6, 3.4)	4.2 (3.3, 5.2)
Zopiclone		3.3 (2.3, 4.3)	6.9 (4.9, 8.8)

BZD: benzodiazepines and z‐drugs; DBP: diastolic blood pressure; SBP: systolic blood pressure. IRSAD: The Index of Relative Socio‐economic Advantage and Disadvantage Advantaged.

Practice IRSAD: an area‐level socioeconomic indicator assigned to practices based on practice postcode. Patients’ IRSAD: an area‐level socioeconomic indicator assigned to patients based on their postcode.

All analyses were conducted on Stata MP 15.1 (StataCorp, Texas, USA), considering the practice as a cluster.

The results from multiple imputed data are presented as the main findings and complete case analyses are provided as supplementary material.

## 3 RESULTS

There were 523,486 regular adult patients (aged ≥18 years) attending 402 Australian general practices with at least one annual visit to the GP each year between 2016 and 2018 and with no records of BZD prescriptions in 2016. Of these, 16,623 (3.2%) were recorded as having been prescribed short‐term BZD and 2532 (0.5%) had an incident long‐term BZD prescription in 2017 (Figure 1).

Table 1 shows that patients recorded as having been exposed to incident short‐term and long‐term BZD were older and had a higher proportion of smokers, patients with sleep issues/insomnia, mental stress or treated with antihypertensive medication than those unexposed to BZD. However, the distribution according to other variables was relatively similar. These patterns were also observed in complete case analyses (Supplementary Table S1).

Table 2 presents crude and adjusted results based on linear regression models and ATE of short‐term and long‐term incident management with BZD on BP. There were slight differences in the crude mean BP of patients exposed and unexposed to BZD. For example, crude mean SBP was 131.2 mmHg for short‐term BZD, 129.8 mmHg for long‐term BZD management, and 130.9 mmHg for those unexposed to BZD. ATE showed that patients managed with short‐term BZD had a slightly higher mean SBP (ATE 0.4; 95% CI 0.1, 0.7) and DBP (ATE 0.5; 95% CI 0.3, 0.7) than those not managed with BZD. On the other hand, mean SBP of patients exposed to long‐term BZD was lower (ATE −1.1; 95% CI −2.0, −0.2), compared with unexposed, but there was almost no difference in the mean DBP of the exposed and unexposed patients. Linear regression models showed lower SBP and DBP among patients managed with long‐term BZD. Findings from complete case analyses showed similar patterns (Supplementary Table S2).

**TABLE 2:** Crude and adjusted analyses^a^ for the use of BZD on systolic and diastolic blood pressure among regular patients attending Australian general practice between 2016 and 2018 (imputed data, total *N* = 523,486)

	*N*	Crude results				Adjusted results			
		Mean	SE	*β*	95% CI	Linear regression		AIPW	
						*β*	95% CI	ATE	95% CI
**Systolic blood pressure**									
No BZD	504,331	130.9	0.0	Ref		Ref		Ref	
Short‐term BZD	16,623	131.2	0.1	0.5	(0.2, 0.8)	0.1	(−0.2, 0.3)	0.4	(0.1, 0.7)
Long‐term BZD	2532	129.8	0.3	−0.8	(−1.4, −0.1)	−1.5	(−2.1, −0.9)	−1.1	(−2.0, −0.2)
**Diastolic blood pressure**									
No BZD	504,331	77.3	0.0	Ref		Ref		Ref	
Short‐term BZD	16,623	77.6	0.1	0.3	(0.1, 0.5)	0.2	(0.1, 0.4)	0.5	(0.3, 0.7)
Long‐term BZD	2532	76.8	0.2	−0.5	(−0.9, −0.1)	−0.5	(−0.9, −0.2)	−0.1	(−0.8, 0.5)

BZD: benzodiazepines and z‐drugs; SE: standard error; *β*: regression coefficient; AIPW: augmented inverse‐probability weighting; ATE: average treatment effect.

^a^ Adjusted for age, sex, rurality, IRSAD, Aboriginal and Torres Strait Islander peoples or not, sleep issues/insomnia, mental stress, diabetes, antihypertensive medication, smoking, baseline systolic blood pressure and baseline diastolic blood pressure.

**FIGURE 2:**
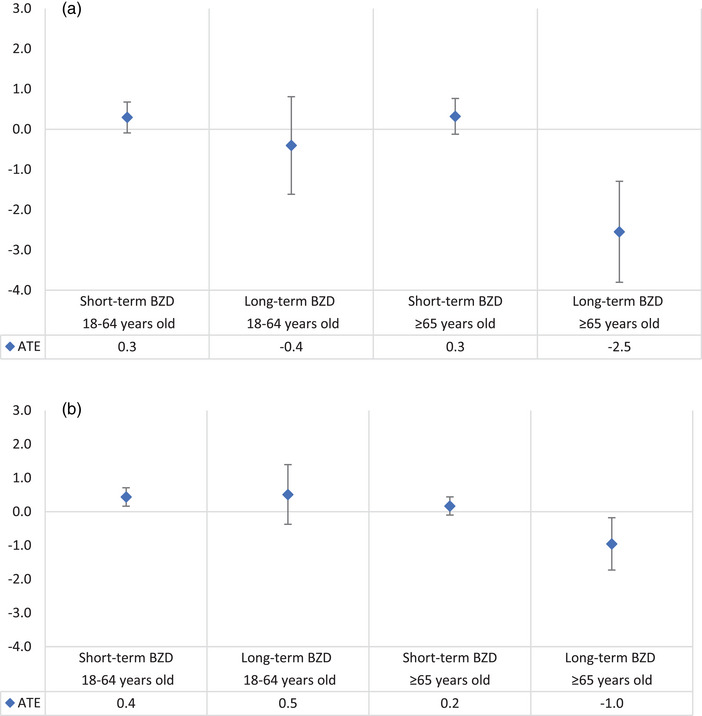
Average treatment effect (ATE) of BZD on systolic blood pressure (a) and diastolic blood pressure (b), both adjusted for age, sex, rurality, IRSAD, Aboriginal and Torres Strait Islander peoples or not, sleep issues/insomnia, mental stress, diabetes, antihypertensive medication, smoking and baseline blood pressure. (Imputed data, total *N* = 523,486). Note. BZD: benzodiazepines and z‐drugs. Vertical lines represent the 95% confidence interval.

Figure 2a and b illustrates the ATE of BZD treatment on BP stratified by age. Short‐term BZD management had a similar effect on SBP or DBP in either age group. However, the BP‐lowering effect of long‐term management with BZD was only observed among those aged 65+ years, with a stronger effect on SBP (ATE −2.5; 95% CI −3.8, −1.3) than DBP (ATE −1.0; 95% CI −1.7, −0.2) when compared to their peers not managed with BZD. Complete case analyses provided similar findings (Supplementary Figure S2a and b).

As BMI was only measured for 41% of the study population, we did not include this variable in primary analyses; however, sensitivity analyses adjusted by BMI provided consistent estimates with the main findings (Supplementary Table S3).

**FIGURE 3:**
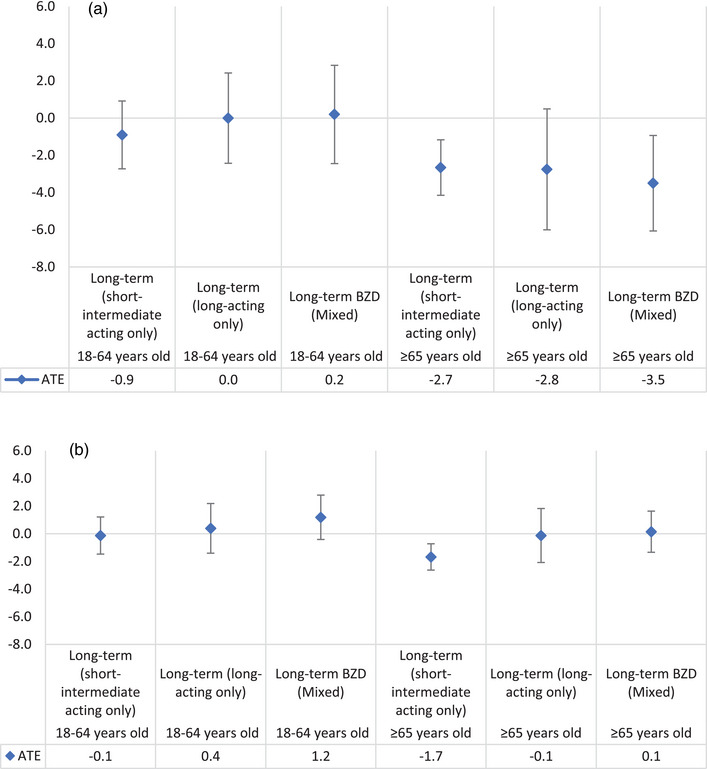
Average treatment effect (ATE) of long‐term BZD on systolic blood pressure (a) and diastolic blood pressure (b), both adjusted for age, sex, rurality, IRSAD, Aboriginal and Torres Strait Islander peoples or not, sleep issues/insomnia, mental stress, diabetes, antihypertensive medication, smoking, and baseline blood pressure (imputed data, total *N* = 506,863). Note. Short‐intermediate acting only: exclusively received short‐intermediate acting BZD in their long‐term episode. Long‐acting only: exclusively received long‐acting BZD in their long‐term episode. Mixed: received a mix of both short‐ and long‐acting BZD in their long‐term episode.

Analyses conducted according to the elimination half‐life of the BZD used for long‐term management (short‐intermediate acting BZD only, long‐acting BZD only, or a mix of both short‐intermediate and long‐acting) showed that, irrespective of the half‐life of BZD prescribed for older patients, all of them were associated with lower SBP when compared to their peers not managed with BZD (Figure 3a). However, the BP‐lowering effect of long‐term management with BZD on DBP among older patients was only observed among those exclusively managed with short‐intermediate acting BZD (Figure 3b). Similar finding were found using complete case analyses (Supplementary Figure S3a and b and Supplementary Table S5). All findings remain consistent with the main findings when we conducted analyses excluding patients with diagnosed sleep apnea (Supplementary Table S4).

## 4 DISCUSSION

In this large cohort study of 523,486 adult patients regularly attending general practices across Australia, we used doubly robust method (AIPW) to compute the ATE of BZD management on BP. Consistent with our original hypothesis, we found long‐term management with BZD was associated with lower SBP and DBP among older patients but not among adults, compared with patients not managed with BZD. The BP‐lowering effect on SBP was irrespective of the half‐life of these drugs, although for DBP it was more evident for long‐term management with short‐intermediate acting BZD. On the other hand, a slightly higher SBP and DBP was observed among all patients exposed to short‐term BZD, irrespective of their age. These findings suggest the BP‐lowering effect of long‐term management with BZD is an additional factor that may contribute to the risk of falls and accidents in this vulnerable population (de Vries et al., 2013).

### 4.1 Comparison with other studies

We found lower BP levels among older patients managed with BZD for long‐term, consistent with Israeli (Mendelson et al., 2017) and Irish (Rivasi et al., 2020) retrospective studies. These studies reported that the exposure to long‐term BZD was associated with a lower BP among patients aged 60+ years. On the contrary, a retrospective study from Belgium reported that long‐term use of short or intermediate half‐life BZD among 1272 patients with insomnia was associated with increased BP (Hein et al., 2019). However, long‐term use of long‐acting BZD was associated with a slightly lower risk of hypertension in that study (Hein et al., 2019).

We found an opposite effect of short‐term and long‐term management with BZD on BP. One of the potential reasons for these varying results could be that in the short‐term management (which is a recommended practice) patients are mostly prescribed one type of BZD and a smaller dose. However, in the long‐term management, patients could be prescribed different types of BZD, and longer use of these medications may also increase tolerance leading to higher dose, which could have an accumulative effect on vasodilation and vasorelaxation, eventually on BP (Crestani et al., 2001; Kagota et al., 2021). Although we did not have information on the change of dose over the course of the long‐term management, we explored what types of drugs were prescribed in the long‐term episode. For example, in our study, 94% of patients with a short‐term BZD management in 2017 were prescribed only one type of BZD. On the contrary, among the patients with a long‐term BZD management in 2017, 49% were exposed to short‐intermediate acting BZD, 23% were exposed to long‐acting BZD, and 28% were prescribed a combination of both short‐ and long‐acting BZD.

Apart from the increasing tolerance due to long‐term management with BZD, the hypotensive effect of long‐term BZD prescriptions observed only among older patients could also be due to alteration in the pharmacokinetics of these drugs with increasing age, leading to impaired clearance, and higher plasma concentration of BZD and its metabolites (Madhusoodanan & Bogunovic, 2004). Indeed, the elimination half‐life of BZD, particularly diazepam, increases with age and is almost double among older than younger people (Herman & Wilkinson, 1996). In addition, the vasodilatory and muscle relaxant properties of the BZDs have also been documented (Colussi et al., 2017; Kagota et al., 2021), which could be another reason for reduction in blood pressure after long‐term exposure to these medications.

### 4.2 Strengths and limitations

Major strengths of this paper are the large number of patients attending general practices across Australia and the routinely collection of data by general practitioners, which reduce recall bias. Also, practices included in the MedicineInsight database are reflective of Australian national data, as patients within MedicineInsight were similar in terms of age, sex, and socioeconomic position than the national Medicare Benefits Schedule (MBS) database of all Australians visiting a GP in 2019–2020 (NPS MedicineWise, 2021). In addition, use of doubly robust method is a strength of this paper, because correct specification of either the treatment model or the outcome model could produce unbiased estimates. The consistency in our findings using different methods and analyses conducted on complete case and imputed data also shows the robustness of the findings.

However, some limitations need to be recognized. First, using prescription data may not be a true reflection of actual BZD use, because we do not know whether these prescriptions were filled and consumed by the patients. Also, BZD prescription included data only from practices included in the MedicineInsight database, so scripts obtained from other sources (such as practices outside MedicineInsight, specialists or hospitals) are not included in the study, which might lead to misclassification, especially for long‐term management. In addition, we did not have information on the medication dose, therefore, could not explore the changes of BZD dose on BP overtime. In terms of unmeasured confounding, although we did not have individual‐level information on socioeconomic position and education, we adjusted for an area‐level measure of socioeconomic status.

## 5 CONCLUSION

We observed lower SBP and DBP among older patients who were managed with BZD for long‐term. These findings suggest that the BP‐lowering effect could be one of the potential reasons why BZD use has been implicated in the increased risk of dizziness, falls and fractures in older people. As these medications are traditionally prescribed to patients with anxiety or sleep issues (Begum et al., 2021), nonpharmacological treatment options should be available and accessible to prevent long‐term BZD use and the risk of falls in old age.

## AUTHOR CONTRIBUTIONS

MB conducted the analyses, interpreted the findings, and prepared the draft. NS, DGC, and CB acquired the data and contributed to conceptualizing and designing, interpreting the findings, critically reviewing, and editing the manuscript.

## FUNDING

No funding was involved in this study.

## CONFLICT OF INTEREST STATEMENT

There is no competing interest to declare. This paper has not been presented anywhere.

### ETHICS STATEMENT

The independent MedicineInsight Data Governance Committee approved this study (protocol 2019–029), and it was exempted from ethical review by the Human Research Ethics Committee of The University of Adelaide because of the use of existing and nonidentifiable data.

### PEER REVIEW

The peer review history for this article is available at https://publons.com/publon/10.1002/brb3.2943.

## Supporting information

Supplementary Table S1. Distribution of regular patients who visited Australian general practice between 2016 and 2018 according to sociodemographic and clinical characteristics by management with BZD among those with full data (complete case analyses, total *N* = 427,239).
Supplementary Table S2. Crude and adjusted analyses* for the use of BZD on systolic and diastolic blood pressure among regular patients with full data attending Australian general practice between 2016 and 2018 (complete case analyses, total *N* = 427,239).
Supplementary Table S3. Average treatment effect* (ATE) of short‐ and long‐term BZD on systolic and diastolic blood pressure among regular adult patients attending Australian general practice (2016‐2018), including BMI as a confounder. Sensitivity analyses, *N* = 523,486.
Supplementary Figure S1. Directed acyclic graph (DAG) showing confounding structure.
Supplementary Figure S2. Average treatment effect (ATE) of BZD on systolic blood pressure (a) and diastolic blood pressure (b).
Supplementary Figure S3. Average treatment effect (ATE) of long‐term BZD on systolic blood pressure (a) and diastolic blood pressure (b).
Supplementary Table S4. Sensitivity analyses: Average treatment effect* (ATE) of short‐ and long‐term BZD on systolic and diastolic blood pressure among regular adult patients attending Australian general practice (2016‐2018), excluding patients with sleep apnea. *N* = 518,677.
Supplementary Table S5. Average treatment effect (ATE)* of long‐term BZD on blood pressure by age. Complete case analyses, total *N* = 427,239.

## Data Availability

MedicineInsight data are not publicly available and not owned by the researchers. To access and use this database, an application can be lodged at the MedicineInsight data governance office.
