# Supplementary material for: Impact of long‐term management with sleep medications on blood pressure: An Australian national study
Source: Brain Behav. 2023 Apr 3;13(5):e2943. doi: 10.1002/brb3.2943 (PMC10175978; doi:10.1002/brb3.2943)
Supplement: Supplementary file 1 — Supplementary Table S1. Distribution of regular patients who visited Australian general practice between 2016 and 2018 according to sociodemographic and clinical characteristics by management with BZD among those with full data (complete case analyses, total N = 427,239). Supplementary Table S2. Crude and adjusted analyses* for the use of BZD on systolic and diastolic blood pressure among regular patients with full data attending Australian general practice between 2016 and 2018 (complete case analyses, total N = 427,239). Supplementary Table S3. Average treatment effect* (ATE) of short‐ and long‐term BZD on systolic and diastolic blood pressure among regular adult patients attending Australian general practice (2016‐2018), including BMI as a confounder. Sensitivity analyses, N = 523,486. Supplementary Figure S1. Directed acyclic graph (DAG) showing confounding structure. Supplementary Figure S2. Average treatment effect (ATE) of BZD on systolic blood pressure (a) and diastolic blood pressure (b). Supplementary Figure S3. Average treatment effect (ATE) of long‐term BZD on systolic blood pressure (a) and diastolic blood pressure (b). Supplementary Table S4. Sensitivity analyses: Average treatment effect* (ATE) of short‐ and long‐term BZD on systolic and diastolic blood pressure among regular adult patients attending Australian general practice (2016‐2018), excluding patients with sleep apnea. N = 518,677. Supplementary Table S5. Average treatment effect (ATE)* of long‐term BZD on blood pressure by age. Complete case analyses, total N = 427,239. [file BRB3-13-e2943-s001.docx]

**Impact of long-term management with sleep medications on blood pressure: an Australian national study**

**Running title:** Benzodiazepines and blood pressure

Mumtaz BEGUM^a^

David GONZALEZ-CHICA^a,b^

Carla BERNARDO^a^

Nigel STOCKS^a,c^

^a^ Discipline of General Practice, Adelaide Medical School, Faculty of Health and Medical Science, The University of Adelaide, Australia.

^b^ Adelaide Rural Clinical School, Adelaide Medical School, Faculty of Health and Medical Science, The University of Adelaide, Australia.

^c^Adelaide Institute for Sleep Health: A Flinders Centre of Research Excellence, College of Medicine and Public Health, Flinders University, South Australia, Australia.

**Supplementary Material**

Table S1. Distribution of regular patients who visited Australian general practice between 2016 and 2018 according to sociodemographic and clinical characteristics by management with BZD among those with full data (complete case analyses, total N= 427,239)

|  | No recorded BZD (%) | Recorded short-term BZD (%) | Recorded long-term BZD (%) |
| --- | --- | --- | --- |
|  | n=411,205 | n=14,006 | n=2,028 |
| **Practice Characteristics** |  |  |  |
| Practice IRSAD |  |  |  |
| Advantaged, highest two quintiles | 39.2 | 43.2 | 37.0 |
| Middle | 24.3 | 23.2 | 23.7 |
| Disadvantaged, lowest two quintiles | 36.6 | 33.6 | 39.3 |
| Rurality |  |  |  |
| Major Cities | 57.2 | 60.1 | 55.8 |
| Inner regional | 28.9 | 27.5 | 29.3 |
| Outer/Remote/Very Remote | 13.9 | 12.4 | 14.9 |
| **Patient's characteristics** |  |  |  |
| Sex |  |  |  |
| Male | 43.7 | 35.7 | 40.7 |
| Female | 56.3 | 64.3 | 59.3 |
| Age (years) |  |  |  |
| 18-34 | 9.9 | 7.4 | 5.9 |
| 35-49 | 15.1 | 14.6 | 15.7 |
| 50-64 | 28.9 | 27.9 | 23.1 |
| 65-74 | 24.2 | 25.6 | 22.7 |
| 75+ | 21.8 | 24.5 | 32.6 |
| Aboriginal and/or Torres Strait Islander peoples |  |  |  |
| Neither Aboriginal nor Torres Strait Islander | 81.6 | 82.2 | 82.1 |
| Aboriginal and/or Torres Strait Islander | 1.7 | 2.1 | 2.5 |
| Not stated/not recorded | 16.7 | 15.7 | 15.4 |
| IRSAD |  |  |  |
| Advantaged, highest two quintiles | 38.4 | 41.8 | 35.4 |
| Middle | 23.9 | 23.4 | 23.7 |
| Disadvantaged, lowest two quintiles | 37.7 | 34.8 | 41.0 |
| Sleep issues/insomnia |  |  |  |
| No | 97.8 | 78.6 | 63.8 |
| Yes | 2.2 | 21.4 | 36.2 |
| Mental stress |  |  |  |
| No | 90.8 | 67.5 | 56.3 |
| Yes | 9.2 | 32.5 | 43.7 |
| Diabetes |  |  |  |
| No | 89.3 | 88.4 | 86.9 |
| Yes | 10.7 | 11.6 | 13.1 |
| AHT medications |  |  |  |
| No | 46.4 | 40.4 | 37.1 |
| Yes | 53.6 | 59.6 | 62.9 |
| Smoking status |  |  |  |
| Non-smokers | 56.3 | 52.2 | 44.0 |
| Smoker | 9.2 | 10.9 | 16.8 |
| Ex-smokers | 30.6 | 33.4 | 35.2 |
| Not stated/not recorded | 3.9 | 3.4 | 4.0 |
| Baseline SBP in mmHg (mean±SD) | 131.9±15.5 | 131.9±15.2 | 131.8±15.2 |
| Baseline DBP in mmHg (mean±SD) | 77.5±9.6 | 77.7±9.6 | 77.3±9.9 |
| Post-treatment SBP in mmHg (mean±SD) | 131.8±15.3 | 131.9±17.8 | 130.1±15.1 |
| Post-treatment DBP in mmHg (mean±SD) | 77.2±9.5 | 77.5±11.1 | 76.4±9.7 |
| Temazepam |  | 40.6 | 35.3 |
| Diazepam |  | 38.0 | 29.0 |
| Oxazepam |  | 8.0 | 14.2 |
| Nitrazepam |  | 1.1 | 2.6 |
| Alprazolam |  | 1.6 | 2.8 |
| Lorazepam |  | 2.5 | 3.5 |
| Clonazepam |  | 1.5 | 1.5 |
| Flunitrazepam |  | 0.1 | 0.1 |
| Clobazam |  | 0.1 | 0.1 |
| Midazolam |  | 0.2 | 0.3 |
| Bromazepam |  | 0.2 | 0.3 |
| Zolpidem |  | 2.8 | 3.9 |
| Zopiclone |  | 3.4 | 6.4 |

BZD: benzodiazepines and z-drugs; IRSAD: The Index of Relative Socio-economic Advantage and Disadvantage Advantaged; DBP: diastolic blood pressure; SBP: systolic blood pressure.

Supplementary Table S2. Crude and adjusted analyses* for the use of BZD on systolic and diastolic blood pressure among regular patients with full data attending Australian general practice between 2016 and 2018 (complete case analyses, total N=427,239)

|  | N | Crude results | | | |  |  | | Adjusted results | | | |
| --- | --- | --- | --- | --- | --- | --- | --- | --- | --- | --- | --- | --- |
|  |  | Mean | SD | Difference in crude mean |  |  | Linear regression | | |  | AIPW | |
|  |  |  |  |  |  |  | β | 95% CI | |  | ATE | 95% CI |
| **Systolic blood pressure** |  |  |  |  |  |  |  |  | |  |  |  |
| No BZD | 411,205 | 131.8 | 15.3 | Ref |  |  | Ref |  | |  | Ref |  |
| Short-term BZD | 14,006 | 132.0 | 17.9 | 0.2 |  |  | 0.2 | -0.1 to 0.4 | |  | 0.5 | 0.2 to 0.8 |
| Long-term BZD | 2,028 | 130.1 | 15.1 | -1.6 |  |  | -1.8 | -2.4 to -1.2 | |  | -1.0 | -1.9 to -0.1 |
| **Diastolic blood pressure** |  |  |  |  |  |  |  |  | |  |  |  |
| No BZD | 411,205 | 77.2 | 9.6 | Ref |  |  | Ref |  | |  | Ref |  |
| Short-term BZD | 14,006 | 77.5 | 11.1 | 0.3 |  |  | 0.2 | 0.0 to 0.4 | |  | 0.4 | 0.2 to 0.6 |
| Long-term BZD | 2,028 | 76.4 | 9.8 | -0.8 |  |  | -0.7 | -1.0 to -0.3 | |  | -0.1 | -0.7 to 0.5 |

BZD: benzodiazepines and z-drugs; SD: standard deviation; β: regression coefficient; AIPW: augmented inverse-probability weighting; ATE: average treatment effect. * Adjusted for age, sex, rurality, IRSAD, Aboriginal and Torres Strait Islander peoples or not, sleep issues/insomnia, mental stress, diabetes, antihypertensive medication, smoking, baseline systolic blood pressure and baseline diastolic blood pressure.

Supplementary Table S3. Average treatment effect* (ATE) of short and long-term BZD on systolic and diastolic blood pressure among regular adult patients attending Australian general practice (2016-2018), including BMI as a confounder. Sensitivy analyses, N = 523,486

|  | **Systolic Blood Pressure** | | | **Diastolic blood pressure** | | |
| --- | --- | --- | --- | --- | --- | --- |
|  | **ATE** | **95 % CI** | | **ATE** | **95 % CI** | |
| No recorded BZD | Ref |  |  | Ref |  |  |
| Recorded short-term BZD use | 0.4 | 0.0 | 0.9 | 0.3 | 0.1 | 0.6 |
| Recorded long-term BZD use | -1.2 | -2.5 | 0.0 | -0.2 | -1.0 | 0.6 |

BZD: benzodiazepines and z-drugs; BMI: body mass index; CI: confidence interval. * Adjusted for age, sex, rurality, IRSAD, Aboriginal and Torres Strait Islander peoples or not, sleep issues/insomnia, mental stress, diabetes, antihypertensive medication, smoking and baseline blood pressure and BMI.

Supplementary Table S4: Sensitivity analyses: Average treatment effect* (ATE) of short and long-term BZD on systolic and diastolic blood pressure among regular adult patients attending Australian general practice (2016-2018), excluding patients with sleep apnoea. N=518,677

|  | **Systolic Blood Pressure** | | | **Diastolic blood pressure** | | |
| --- | --- | --- | --- | --- | --- | --- |
|  | **ATE** | **95 % CI** | | **ATE** | **95 % CI** | |
| **Regular patients (all patients)** |  |  |  |  |  |  |
| No-BZD | Ref |  |  | Ref |  |  |
| Short term BZD | 0.4 | 0.1 | 0.7 | 0.3 | 0.1 | 0.5 |
| Long-term BZD | -0.9 | -1.9 | 0.0 | -0.1 | -0.7 | 0.5 |
| **Regular patients (18-64 years old)** |  |  |  |  |  |  |
| No-BZD | Ref |  |  | Ref |  |  |
| Short term BZD | 0.5 | 0.1 | 0.9 | 0.4 | 0.1 | 0.7 |
| Long-term BZD | 0.1 | -1.2 | 1.5 | 0.9 | 0.0 | 1.9 |
| **Regular patients (≥65 years old)** |  |  |  |  |  |  |
| No-BZD | Ref |  |  | Ref |  |  |
| Short term BZD | 0.4 | -0.1 | 0.8 | 0.1 | -0.2 | 0.4 |
| Long-term BZD | -2.3 | -3.6 | -1.1 | -1.2 | -1.9 | -0.5 |
| **Regular patients (18-64 years old)** |  |  |  |  |  |  |
| Long-term (short-intermediate acting only) | -1.6 | -3.6 | 0.3 | -0.5 | -1.9 | 1.0 |
| Long-term (long-acting only) | 1.1 | -1.7 | 4.0 | 0.8 | -0.7 | 2.4 |
| Long-term BZD (Mixed) | 1.4 | -1.3 | 4.1 | 2.4 | 0.7 | 4.1 |
| **Regular patients (≥65 years old)** |  |  |  |  |  |  |
| Long-term (short-intermediate acting only) | -2.2 | -3.7 | -0.8 | -1.7 | -2.5 | -0.9 |
| Long-term (long-acting only) | -3.4 | -7.0 | 0.1 | 0.5 | -1.7 | 2.7 |
| Long-term BZD (Mixed) | -4.0 | -7.0 | -1.1 | -0.3 | -1.9 | 1.4 |

BZD: benzodiazepines and z-drugs; CI: confidence interval.

* Adjusted for age, sex, rurality, IRSAD, Aboriginal and Torres Strait Islander peoples or not, sleep issues/insomnia, mental stress, diabetes, antihypertensive medication, smoking and baseline blood pressure


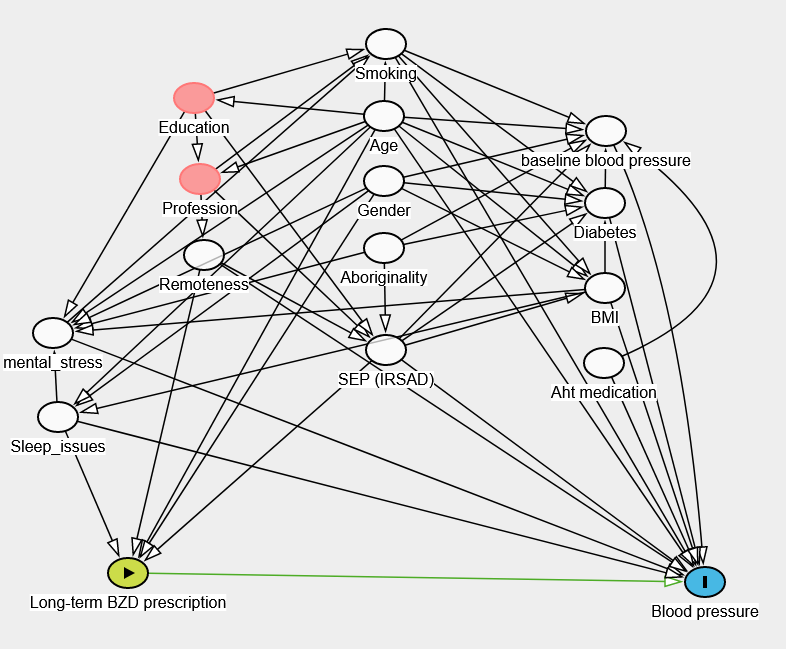


Supplementary Figure S1: Directed Acyclic Graph (DAG) showing confounding structure

Note. BZD: benzodiazepines and z-drugs. Vertical lines represent the 95% confidence interval.

Supplementary Figure S2. Average treatment effect (ATE) of BZD on systolic blood pressure (Figure S2a) and diastolic blood pressure (Figure S2b), both adjusted for age, sex, rurality, IRSAD, Aboriginal and Torres Strait Islander peoples or not, sleep issues/insomnia, mental stress, diabetes, antihypertensive medication, smoking and baseline blood pressure. Complete case analyses, total N=427,239.

Note. Short-acting only: exclusively received short-acting BZD in their long-term episode. Long-acting only: exclusively received long-acting BZD in their long-term episode. Mixed: received a mix of both short and long-acting BZD in their long-term episode.

Supplementray Figure S3. Average treatment effect (ATE) of long-term BZD on systolic blood pressure (Figure S3a) and diastolic blood pressure (Figure S3b), both adjusted for age, sex, rurality, IRSAD, Aboriginal and Torres Strait Islander peoples or not, sleep issues/insomnia, mental stress, diabetes, antihypertensive medication, smoking and baseline blood pressure. Complete case analyses, total N=427,239.

Supplementary Table S5. Average treatment effect (ATE)* of long-term BZD on blood pressure by age. Complete case analyses, total N=427,239.

|  |  | **Systolic Blood Pressure** | | | | | | |  | | **Diastolic Blood Pressure** | | | | | | |
| --- | --- | --- | --- | --- | --- | --- | --- | --- | --- | --- | --- | --- | --- | --- | --- | --- | --- |
|  | N | Crude Mean | SD | Difference  in crude mean | ATE | 95%CI | | N | | Crude Mean | | SD | Difference in crude mean | ATE | 95%CI | |  |
| **18-64 years old** |  |  |  |  |  |  |  |  | |  | |  |  |  |  |  |  |
| No BZD | 214,880 | 127.8 | 15.2 | Ref | Ref |  |  | 214,880 | | 79.2 | | 9.7 | Ref | Ref |  |  |  |
| Long-term BZD (Short-acting only) | 337 | 126.8 | 13.6 | -1.0 | -1.7 | -3.5 | 0.2 | 337 | | 79.2 | | 9.6 | 0.0 | -0.3 | -1.8 | 1.1 |  |
| Long-term BZD (Long-acting only) | 271 | 127.8 | 15.2 | 0.0 | 1.1 | -1.7 | 3.9 | 271 | | 80.5 | | 9.8 | 1.4 | 0.8 | -0.8 | 2.3 |  |
| Long-term BZD (Mixed) | 293 | 127 | 15.2 | -0.8 | 1.3 | -1.3 | 4.0 | 293 | | 80.3 | | 9.0 | 1.1 | 2.4 | 0.8 | 4.1 |  |
| **65+ years old** |  |  |  |  |  |  |  |  | |  | |  |  |  |  |  |  |
| No BZD | 196,325 | 136.1 | 14.3 | Ref | Ref |  |  | 196,325 | | 75.1 | | 9.0 | Ref | Ref |  |  |  |
| Long-term BZD (Short-acting only) | 745 | 132.3 | 15 | -3.7 | -2.2 | -3.7 | -0.8 | 745 | | 73.1 | | 9.0 | -2.0 | -1.8 | -2.5 | -1.0 |  |
| Long-term BZD (Long-acting only) | 124 | 134.1 | 14.8 | -2.0 | -3.3 | -6.8 | 0.2 | 124 | | 75.1 | | 9.9 | -0.1 | 0.2 | -1.9 | 2.2 |  |
| Long-term BZD (Mixed) | 258 | 132.2 | 15.4 | -3.8 | -4.0 | -6.8 | -1.1 | 258 | | 74.5 | | 9.1 | -0.6 | -0.2 | -1.8 | 1.4 |  |

BZD: benzodiazepines and z-drugs; SD (standard deviation); CI: confidence interval. *Adjusted for age, sex, rurality, IRSAD, Aboriginal and Torres Strait Islander peoples or not, sleep issues/insomnia, mental stress, diabetes, antihypertensive medication, smoking and baseline blood pressure.
